# Beyond pay and balance: organizational support as a driver of professional development across generations in the Chilean mining industry

**DOI:** 10.3389/fpsyg.2026.1779397

**Published:** 2026-05-20

**Authors:** Jorge Serrano-Malebrán, Carlos Molina, Paola von-Bischoffshausen

**Affiliations:** 1 Facultad de Ingeniería y Negocios, Universidad de Las Américas, Providencia, Santiago, Chile; 2 Facultad de Economía y Administración, Centro de Emprendimiento y de la Pyme, Universidad Católica del Norte, Antofagasta, Chile; 3 Departamento de Auditoría, Contabilidad y Control de Gestión, Universidad Católica del Norte, Antofagasta, Chile

**Keywords:** generational differences, pay satisfaction, perceived organizational support, professional development, work–life balance

## Abstract

**Introduction:**

Professional development is increasingly recognized as a key component of sustainable careers, particularly in demanding sectors such as mining. However, limited evidence exists on how perceived organizational support relates to professional development, pay satisfaction, and work–life balance across generational cohorts in this context.

**Methods:**

This study examined the relationship between Perceived Organizational Support (POS) and Professional Development (PD) in Chile’ s mining sector and explored whether POS was associated with Pay Satisfaction (PS) and Work–Life Balance (WLB). It also assessed generational differences across Generations X, Y, and Z. The model was estimated using partial least squares structural equation modeling (PLS-SEM) with a sample of 203 employees.

**Results:**

The results showed that POS was positively associated with PD, as well as with WLB and PS. In contrast, neither PS nor WLB was significantly associated with PD. Multigroup analysis suggested that the relationship between POS and pay satisfaction may be stronger for Generation X than for Generation Z.

**Discussion:**

These findings provide empirical evidence of a relationship that remains underexplored in extractive industries in developing-country contexts and underscore the relevance of POS as a structuring resource for sustainable career trajectories and professional development processes. From a practical standpoint, the study suggests the potential value of considering generational differences in talent management strategies. Methodological limitations are acknowledged, and directions for future research are proposed, including longitudinal designs and comparative sectoral approaches.

## 1 Introduction

Professional development is understood as a dynamic, continuous, and self-regulated process through which individuals shape their career trajectories over time by integrating personal goals, accumulated learning, and changing contextual conditions (Barhate and Dirani, 2022; Savickas, 2012). This perspective departs from traditional linear career models promoted exclusively by organizations and emphasizes individuals’ active role in managing and planning their professional development (Barhate and Dirani, 2022; De Vos and Van der Heijden, 2017). This shift reflects labor market contexts characterized by uncertainty, intensified competition for talent, and emerging generational demands (Wu et al., 2023). Accordingly, scholarly interest has grown in understanding how organizational conditions influence employees’ career choices.

Within this new paradigm, the concept of sustainable careers has gained relevance. Sustainable careers highlight continuous employability, psychological wellbeing, and value congruence as key pillars for building resilient career paths in dynamic environments (De Vos et al., 2011; De Vos and Van der Heijden, 2017; Spurk et al., 2019). In this framework, perceived organizational support—defined as employees’ perceptions that the organization values their contributions and cares about their wellbeing—has been identified as a robust predictor of job satisfaction, talent retention, and professional growth by fostering conditions that enable sustained learning, career planning, and professional development. Such support may also enhance work–life balance through flexible arrangements, institutional support, and stress reduction, and may shape pay-related perceptions when compensation practices are viewed as equitable and transparent (Barhate and Dirani, 2022; Colquitt et al., 2001; Masuda et al., 2017).

Pay satisfaction is a frequently underestimated yet increasingly relevant factor (Currall et al., 2005). Pay satisfaction refers not only to the amount received, but also to perceived fairness and equity in compensation and the perceived alignment among effort, performance, and rewards. Recent studies suggest that pay satisfaction significantly influences retention, perceptions of organizational justice, and professional commitment, particularly in sectors where shortages of qualified talent heighten turnover risk (Barhate and Dirani, 2022; Masuda et al., 2017).

In parallel, the literature has begun to examine generational differences as a key lens for understanding variations in perceptions, values, and priorities regarding professional development and working conditions (Barhate and Dirani, 2022). Whereas Generation X tends to value stability and institutional career progression, Generations Y and Z often prioritize continuous learning, flexibility, purpose, and value congruence with their work environments. These differences suggest that a one-size-fits-all approach to talent management may be ineffective if cohort-specific characteristics are not considered, particularly when designing professional development strategies.

Despite the growing body of evidence on these constructs, important research gaps remain. Specifically, few studies have simultaneously examined perceived organizational support, work–life balance, and pay satisfaction in relation to professional development while explicitly incorporating a comparative generational perspective. Most existing studies address these variables in isolation, without assessing whether the strength of these relationships varies across age groups or career stages (Barhate and Dirani, 2022). This gap is especially critical in sectors such as mining, which are characterized by demanding working conditions, high turnover, particularly among Generation Z workers (Wulandari et al., 2023), and the coexistence of multiple generations within the same productive environment. In contexts such as the Antofagasta Region in Chile, a strategic hub for global mining, retaining qualified talent has become an organizational priority, requiring a deeper understanding of the factors that shape employees’ decisions to remain and develop professionally within their organizations.

Against this backdrop, this study addresses the following research question: How does perceived organizational support relate to professional development, work–life balance, and pay satisfaction across Generations X, Y, and Z in the Chilean mining industry? To answer this question, we adopted a quantitative, explanatory design and estimated the proposed direct relationships using structural equation modeling (PLS-SEM) based on an online survey of mining industry workers in Chile. In addition, a multigroup analysis was conducted to explore whether these structural relationships differed across generational cohorts. This contextualized approach captures the key features of the labor environment and sector-specific dynamics in industries of national strategic relevance. Thus, this study aims to provide empirical evidence addressing an identified theoretical gap and offer actionable insights for differentiated talent management that considers the specific needs and motivations of each generation.

## 2 Literature review

### 2.1 Social exchange theory

Blau (1964) posits that exchange relationships are causally connected, such that the quality of the social exchange relationship between parties shapes subsequent exchange processes. In other words, SET suggests that when an organization addresses employees’ psychological and emotional needs, employees become more committed to organizational goals and, as a result, are more likely to enhance their performance (Cropanzano and Mitchell, 2005). In this study, SET serves as an overarching theoretical lens linking Perceived Organizational Support (POS), Work–Life Balance (WLB), and Pay Satisfaction (PS) to Professional Development (PD).

### 2.2 Professional development

Professional development is defined as a continuous, ongoing process of acquiring and updating knowledge, skills, and competencies through which individuals improve their performance, strengthen their adaptive capacity, and enhance their career prospects over time (Creta and Gross, 2020; Obedgiu, 2017). Unlike initial training, professional development is commonly conceptualized as lifelong learning, integrating both formal and informal experiences through which workers cultivate a growth mindset that supports sustainable career outcomes (Van der Heijden et al., 2020). This concept is further enriched by perspectives from career development, employability, and sustainable careers, which recognize the shared responsibility between the individual and the organization in managing learning and professional growth across the working lifespan (De Vos and Van der Heijden, 2017; Van der Heijden et al., 2020). Moreover, this perspective aligns closely with the Social Exchange Theory (SET): when organizations invest in employees’ professional development, employees may reciprocate through stronger commitment, higher performance, and greater retention intentions (Eisenberger et al., 1986; Putra et al., 2024). In this sense, professional development can be viewed as a form of social exchange “currency” that reinforces the psychological bond between the employee and the organization, fostering more stable and sustainable employment relationships.

Recent research highlights the individual’s active role in professional development, which involves interpreting experiences, constructing personal narratives of progress, and making decisions that respond to changing contexts (Barhate and Dirani, 2022; Savickas, 2012). From this perspective, career adaptability—the capacity to cope with occupational changes through proactive attitudes, competencies, and behaviors—has been positioned as a key psychosocial resource for sustaining professional development processes and resilient and satisfying career trajectories (Guan et al., 2015; Rasheed et al., 2020). Finally, substantial empirical evidence links perceptions of career development—often shaped by organizational professional development opportunities—to favorable organizational outcomes, such as higher job satisfaction, stronger organizational commitment, and lower turnover intentions (Barthauer et al., 2020). Within the professional development lens, these perceptions reflect ongoing learning experiences and perceived organizational support throughout the career trajectory, which not only promotes positive attitudinal outcomes but also strengthens the competencies and adaptability required to navigate dynamic environments (Creta and Gross, 2020; Putra et al., 2024). Taken together, these findings underscore the need to implement professional development support policies as an integral component of talent management strategies in highly demanding work settings, such as mining. Because professional development refers to employees’ perceptions of growth, improvement, and learning over time, it is likely to be especially sensitive to organizational signals that directly communicate recognition, support, and investment in employee development.

### 2.3 Perceived organizational support

From the perspective of Organizational Support Theory (OST), Perceived Organizational Support (POS) is defined as employees’ general belief regarding the extent to which the organization values their contributions and cares about their wellbeing (Eisenberger et al., 1986; Rhoades and Eisenberger, 2002). POS has been consistently linked to key work-related outcomes, including job satisfaction, organizational commitment, and retention intention (Barhate and Dirani, 2022).

POS functions as a psychosocial resource that strengthens work resilience and career adaptability, particularly when expressed through development opportunities, explicit recognition, and emotional support (Barhate and Dirani, 2022). Evidence suggests that supportive organizational environments enhance employees’ self-efficacy, affective commitment, and perceived professional growth (Johnston, 2018). Moreover, POS contributes to sustainable career trajectories by fostering contexts in which learning, fairness, and career planning are central elements of the work experience (Barhate and Dirani, 2022). More broadly, when organizations demonstrate support and kindness toward employees, it tends to translate into positive individual and organizational outcomes (Liu et al., 2025). In this sense, POS may be expected to show a particularly close relationship with professional development, as it directly signals that the organization values employees’ contributions and supports their long-term growth. Based on this evidence, we propose the following hypothesis:

H1: Perceived Organizational Support (POS) is positively and directly related to Professional Development (PD).

### 2.4 Work–life balance

Work–life balance (WLB) has emerged as a core component of strategic human resource management, integrating employees’ physical, mental, and social wellbeing with their capacity to pursue sustainable professional development. Operationally, WLB is reflected in the effective reconciliation of work and personal responsibilities, as well as in perceptions of an environment that supports both performance and emotional balance (Rajagopal et al., 2024).

In organizational settings that promote flexible scheduling, respect for personal time, and access to institutional support, employees tend to report lower stress levels and a greater willingness to commit to long-term career trajectories (Masuda et al., 2017). WLB is also strengthened by factors such as pay equity, access to resources, and job autonomy, which can enhance perceptions of professional development (Barhate and Dirani, 2022; Rajagopal et al., 2024; Rodríguez-Sánchez et al., 2020). From the perspective of Social Exchange Theory (SET), WLB also entails a bidirectional dynamic: employees focus on achieving organizational goals while benefiting from work–family policies that enable them to balance work and family demands.

WLB is further shaped by perceived organizational support (POS). In contexts where flexibility, respect for personal time, and institutional support are promoted, employees report greater overall wellbeing, lower stress, and a stronger willingness to commit to long-term career paths (Barhate and Dirani, 2022; Masuda et al., 2017).

Empirical evidence indicates that when employees perceive a high level of work–life balance, their job satisfaction increases, along with their sense of efficacy at work and in their personal lives. This condition is positively associated with envisioning a coherent and sustainable career trajectory (Rajagopal et al., 2024; Rodríguez-Sánchez et al., 2020). Accordingly, WLB can be understood as a relevant organizational condition that may be associated with employees’ professional development perceptions. Moreover, WLB appears to operate differently across generations; while older workers often prioritize stability and predictability, younger cohorts tend to value autonomy, flexibility, and learning opportunities. These differences shape how organizational efforts to improve work–life balance are perceived and, consequently, how such efforts influence commitment and professional development (Barhate and Dirani, 2022). In particular, Badaruddin et al. (2024) argue that a balanced work environment increases employees’ willingness to engage in professional development activities, strengthening their technical and managerial competencies and contributing to more satisfying and sustainable career trajectories. Based on the above, we propose the following hypothesis:

H2: Perceived Organizational Support (POS) has a positive and direct effect on Work–Life Balance (WLB).

H4: Work–Life Balance (WLB) is positively and directly related to Professional Development (PD).

### 2.5 Pay satisfaction

Pay satisfaction, defined as an individual’s subjective evaluation of the fairness and adequacy of monetary compensation relative to perceived effort and comparisons with relevant referents, constitutes a key dimension in organizational management (Currall et al., 2005). This evaluative judgment reflects not only personal expectations but is also consistently associated with critical work attitudes, such as organizational commitment, intrinsic motivation, and intention to stay, all of which are relevant indicators of sustainable career trajectories (Masuda et al., 2017). From a Social Exchange Theory (SET) perspective, pay satisfaction is rooted in the exchange relationship between employee and employer, whereby employees provide time and effort in return for financial compensation (Adriaans et al., 2023).

Similarly, perceptions of distributive fairness—particularly when employees perceive a fair correspondence between effort and reward—can reinforce perceived organizational support by signaling that the organization tangibly cares for its employees (Ordu and Sarı, 2022).

When employees perceive their pay as fair, proportional to their contributions, and aligned with internal merit standards, they tend to display higher involvement, greater discretionary effort, and stronger affective bonds with the organization (Colquitt et al., 2001). Similarly, pay practices perceived as transparent and merit-based not only increase job satisfaction but also encourage career decisions oriented toward internal development, thereby strengthening talent retention and the construction of longer organizational career paths (Masuda et al., 2017). Additionally, Singh and Loncar (2010) suggest that pay satisfaction may encourage employees’ willingness to invest effort in learning activities within the organization, particularly when pay is perceived as recognition of skill development and performance.

This relationship is especially important in industries marked by high turnover or shortages of specialized profiles, where perceptions of pay equity may tip the balance in decisions to remain or move. Moreover, significant generational differences have been documented in the valuation of compensation. Generation X tends to prioritize financial stability, whereas Generation Y places greater emphasis on balancing pay and work purpose. In turn, Generation Z prioritizes economic income, opportunities for formative experiences, and flexible work environments (Barhate and Dirani, 2022). Based on the above, we propose the following hypotheses:

H3: Perceived Organizational Support (POS) is positively and directly related to Pay Satisfaction (PS).

H5: Pay Satisfaction (PS) is positively and directly related to Professional Development (PD).

### 2.6 Generational differences

Generational cohorts may shape how employees interpret and value organizational support, work–life balance, compensation, and professional development opportunities. Research suggests that younger generations place particular emphasis on continuous learning, flexibility, and professional growth, whereas older cohorts tend to value stability, structured progression, and institutional recognition more strongly (Barhate and Dirani, 2022; Kupczyk et al., 2024).

Age and generational cohorts may also shape how employees interpret and value organizational support. For instance, research suggests that younger generations place particular emphasis on continuous learning, flexibility, and professional development; consequently, their expectations regarding organizational support may differ from those of earlier cohorts (Barhate and Dirani, 2022). With respect to work–family balance, Generation X is often characterized by a stronger work focus, whereas Generations Y and Z prioritize maintaining a balance between work and personal life (Kupczyk et al., 2024). In other words, Generation X may view work as a central life component, whereas Generations Y and Z are more likely to see work as a means to secure the resources needed to pursue personal goals.

Although all generations value growth opportunities, their priorities and approaches to professional development differ. Generation X tends to emphasize stability, linear progression, and the accumulation of experience, whereas Generations Y (Millennials) and Z place greater value on flexibility, continuous learning, and aligning professional growth with personal wellbeing (Salvadorinho et al., 2026). In particular, younger cohorts appreciate work environments that promote work–life balance and opportunities for training, mentoring, and career paths that enable rapid learning and adaptation. This suggests that professional development policies should be tailored to these diverse expectations to attract and retain talent in dynamic organizational contexts (Fuchs et al., 2024). Based on the above, we propose the following hypothesis:

H6: The structural relationships among Perceived Organizational Support (POS), Work–Life Balance (WLB), Pay Satisfaction (PS), and Professional Development (PD) differ across Generations X, Y, and Z.

**Figure 1:**
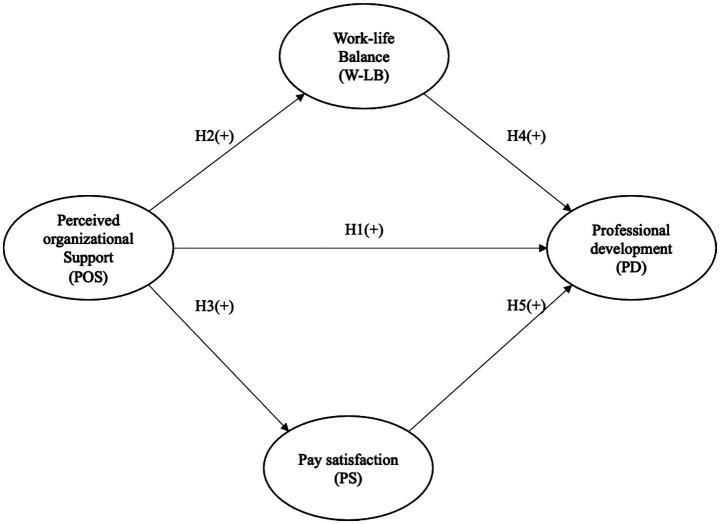
Proposed model.

The proposed model (Figure 1) examines how Perceived Organizational Support (POS) is related to workers’ Professional Development (PD) in Chile’s mining sector and also assesses its direct relationships with Work–Life Balance (WLB) and Pay Satisfaction (PS). Specifically, POS is expected to have a positive direct effect on PD (H1), WLB (H2), and PS (H3). In turn, both WLB (H4) and PS (H5) are expected to be positively associated with PD, thereby integrating a model that links organizational conditions, perceived wellbeing, and professional development.

In addition, a comparative generational perspective was incorporated to explore whether these structural relationships differed across Generations X, Y, and Z. This enables the model to capture variations in perceptions and expectations regarding the organizational environment and professional development opportunities as a function of life stage and cohort membership. Overall, this approach aims to provide a more comprehensive understanding of the factors that shape sustainable careers in age-diverse organizations, particularly in high-demand work contexts.

## 3 Methodology

### 3.1 Population and sample

The study’s target population comprised active employees working in mining sector companies in the Antofagasta Region, Chile, an area that accounts for approximately 53% of national mining employment (Consejo Minero and Fundación Chile, 2023). Participants were drawn from Generations X (born 1965–1980), Y (1981–1996), and Z (1997 onward). These cutoff points were adopted because they are widely used in generational research and allow comparability with prior studies. In addition, these cohort boundaries have also been used in recent Chilean reports and analyses (Del Solar and Fernández, 2024; Lampert, 2025). In this study, they are treated as analytical categories rather than as fixed or uniquely Chilean sociocultural boundaries. A non-probability convenience sampling strategy was used due to limited access to the target population.

The minimum sample size was calculated using G*Power (Faul et al., 2007) 3.1 with a significance level of 0.05, statistical power of 0.95, and a medium effect size (*f*^2^ = 0.15), yielding a minimum requirement of 138 observations. To ensure greater stability of the estimates and enable multigroup analyses, we collected 203 valid responses. Data were gathered between August and October 2024 using a self-administered questionnaire distributed online through Google Forms. The survey link and QR codes were disseminated through collaborating organizations with prior institutional authorization. Because the survey was distributed through organizational channels, the exact number of employees reached could not be established; therefore, a response rate could not be calculated, and information on non-respondents was not available to assess potential non-response bias. Participation was voluntary and anonymous. The study was approved by the Ethics Committee of the Universidad Católica del Norte.

Regarding sample characteristics, 61% of the participants identified as male and 39% as female. The generational distribution was as follows: 22% Generation X, 47% Generation Y, and 31% Generation Z. In terms of educational attainment, 58% reported technical or professional training and 42% reported university or postgraduate education. In terms of job roles, 63% reported operational positions, while 37% held supervisory or leadership roles.

### 3.2 Measurement scales

Study variables were measured using previously validated instruments from the literature, with 5-point Likert-type scales. All scales were adapted to the Chilean mining work context while preserving their conceptual and semantic equivalence. Perceived Organizational Support (POS) was assessed using the scale developed by Eisenberger et al. (1986), which captures employees’ perceptions of the extent to which the organization values their contributions and cares about their wellbeing. Responses were recorded on a 5-point Likert scale ranging from 1 (“strongly disagree”) to 5 (“strongly agree”). A sample item is: “The organization seriously considers my goals and values.”

Professional Development (PD) was measured using the Professional Development Short Scale proposed by Mourão et al. (2022), which assesses employees’ perceived current professional development. In the original study, the scale was specified as an agreement-based measure. Accordingly, in this study, the items were assessed using a 5-point Likert agreement scale ranging from 1 (“strongly disagree”) to 5 (“strongly agree”), adapted to maintain consistency with the overall questionnaire format. A representative item is: “With my current knowledge, I can do my job satisfactorily.” For Work–Life Balance (WLB), we used the scale by Shukla and Srivastava (2016), which assesses the balance between work and non-work demands. Responses were captured on a 5-point scale. Because the instrument includes negatively worded items, these were reverse-coded prior to analysis so that higher scores consistently indicated better work–life balance. An example of a reverse-coded item is: “I have difficulties balancing my work and non-work activities.” Pay Satisfaction (PS) was measured using an adapted version of the scale developed by Heneman and Schwab (1985), organized into four dimensions: pay structure (PST), pay raise (PR), benefits (BF), and pay level (PL). Consistent with its multidimensional nature, PS was modeled as a second-order construct. In the first stage, the four dimensions were estimated as first-order latent variables; in the second stage, the latent variable scores of PST, PR, BF, and PL were used as indicators of the higher-order PS construct. Responses were recorded on a 5-point scale ranging from 1 (“very dissatisfied”) to 5 (“very satisfied”). Example items include: “I am satisfied with my current pay,” “I am satisfied with my benefits package,” “I am satisfied with the company’s pay structure,” and “I am satisfied with my most recent pay raise.”

### 3.3 Statistical procedures

Data analysis was conducted using partial least squares structural equation modeling (PLS-SEM) with SmartPLS4 (Ringle et al., 2022). This technique was selected because of its suitability for models with multiple latent variables, robustness to non-normal data, and effectiveness in studies with moderate sample sizes (Hair et al., 2022). The analysis proceeded in two stages. First, the measurement model was assessed in terms of reliability, convergent validity, and discriminant validity. Second, the structural model was evaluated by estimating the direct relationships among the study constructs.

To examine whether these structural relationships differed across generations, measurement invariance was first assessed using the measurement invariance of composite models (MICOM) procedure available in SmartPLS 4. The results supported compositional invariance for all constructs across the X–Y, X–Z, and Y–Z comparisons, thereby establishing partial measurement invariance and allowing meaningful comparison of structural paths across generational groups. After establishing measurement invariance, group differences were examined using permutation multigroup analysis (Permutation MGA), comparing path coefficients across Generations X, Y, and Z. Given the unequal and relatively modest subgroup sizes, a post-hoc sensitivity analysis was conducted to assess the minimum detectable effect size for the multigroup comparisons. This analysis was used to contextualize the interpretation of the multigroup results.

## 4 Results

### 4.1 Measurement model assessment

We estimated a model comprising three first-order constructs (Perceived Organizational Support, Work–Life Balance, and Professional Development) and one second-order construct (Pay Satisfaction), the latter operationalized through four dimensions: pay structure, pay raise, benefits, and pay level. The second-order construct was modeled using a two-step approach, following the methodological recommendations of Sarstedt et al. (2019). In this procedure, the first-order dimensions (PST, PR, BF, and PL) were first estimated separately, and their latent variable scores were subsequently used as indicators of the higher-order PS construct. This approach enables a more accurate representation of the hierarchical nature of complex constructs such as pay satisfaction.

Table 1 reports the descriptive statistics and bivariate correlations for the study constructs and, to reflect the multidimensional specification of Pay Satisfaction, the first-order dimensions of PS (pay structure, pay raise, benefits, and pay level). Overall, the means ranged from 3.146 to 3.908, while standard deviations ranged from 0.743 to 0.918, indicating a reasonable degree of dispersion across the variables and no obvious evidence of severe floor or ceiling effects. At the bivariate level, Perceived Organizational Support (POS) showed moderate positive correlations with Work–Life Balance (WLB) (*r* = 0.427) and Professional Development (PD) (*r* = 0.646), as well as with the four first-order dimensions of Pay Satisfaction (r ranging from 0.383 to 0.520). WLB was positively correlated with PD (*r* = 0.315) and with the pay satisfaction dimensions (*r* ranging from 0.373 to 0.464). In turn, PD showed positive but comparatively weaker correlations with the pay satisfaction dimensions (*r* ranging from 0.280 to 0.320). The correlations among the four first-order dimensions of Pay Satisfaction were high, as expected, ranging from 0.780 to 0.831, which is consistent with their specification as dimensions of a higher-order construct.

**Table 1:** Descriptive statistics.

Variable		Mean	SD	POS	WLB	PD	PST	PR	BF	PL
POS		3.703	0.743	–						
WLB		3.482	0.842	0.427	–					
PD		3.908	0.779	0.646	0.315	–				
PS	Pay structure (PST)	3.146	0.787	0.504	0.452	0.280	–			
	Pay raise (PR)	3.162	0.884	0.520	0.464	0.320	0.827	–		
	Benefits (BF)	3.390	0.918	0.472	0.373	0.299	0.780	0.784	–	
	Pay level (PL)	3.343	0.890	0.383	0.404	0.290	0.789	0.794	0.831	–

Overall, the measurement model demonstrated adequate reliability and convergent validity. All first-order constructs, the four first-order dimensions of Pay Satisfaction, and the higher-order PS construct exhibited Cronbach’s alpha, composite reliability (ρ_a and ρ_c), and average variance extracted (AVE) values above the recommended thresholds (0.70 for reliability and 0.50 for AVE). This confirms internal consistency and convergent validity across all modeled components (Table 2). In addition, the standardized root mean square residual (SRMR) was 0.082, indicating a borderline level of fit, as it lies slightly above the commonly referenced 0.08 threshold. As a complementary fit index, the normed fit index (NFI) was 0.903, which is above the conventional 0.90 benchmark. Taken together, these results suggest that the model demonstrates acceptable overall fit, although the SRMR indicates that this fit should be interpreted with some caution.

**Table 2:** Reliability and convergent validity.

Construct	Cronbach’s alpha	Composite reliability (ρ_a)	Composite reliability (ρ_c)	AVE
POS	0.884	0.888	0.910	0.591
WLB	0.720	0.779	0.874	0.777
PD	0.829	0.852	0.885	0.659
PS (second-order)	0.942	0.949	0.958	0.852
PST	0.906	0.909	0.927	0.680
PR	0.844	0.847	0.896	0.682
BF	0.908	0.912	0.935	0.783
PL	0.944	0.947	0.960	0.857

Discriminant validity was assessed using the heterotrait–monotrait ratio (HTMT) criterion (Table 3). All HTMT values were below the 0.85 threshold, supporting the discriminant validity of the constructs.

Table 2 reports the reliability and convergent validity results for the first-order constructs, the four first-order dimensions of Pay Satisfaction, and the higher-order PS construct. In the case of PS, the higher-order construct was specified using the latent variable scores of PST, PR, BF, and PL as indicators (Table 3).

**Table 3:** Discriminant validity (HTMT).

Construct	POS	WLB	PD
WLB	0.526		
PD	0.794	0.411	
PS	0.560	0.563	0.415

### 4.2 Structural model assessment

The structural model revealed significant and positive effects of Perceived Organizational Support on Professional Development (*β* = 0.686, *p* < 0.001), on Work–Life Balance (*β* = 0.431, *p* < 0.001), and on Pay Satisfaction (*β* = 0.522, *p* < 0.001). In contrast, neither Pay Satisfaction (*β* = 0.005, *p* = 0.933) nor Work–Life Balance (*β* = 0.033, *p* = 0.609) showed significant direct effects on Professional Development (Table 4), indicating that H4 and H5 were not empirically confirmed. These results suggest that, in this sample, perceived professional development is more strongly associated with organizational support than with broader evaluations of compensation or work–life balance.

**Table 4:** Structural model results and hypothesis testing.

Path	Path coefficient	*p* value	Result
POS - >PD	0.686	0.000	Supported
POS - >WLB	0.431	0.000	Supported
POS - >PS	0.522	0.000	Supported
PS - >PD	0.005	0.933	Not supported
WLB - >PD	0.033	0.609	Not supported

To assess the potential influence of common method bias, full collinearity VIF values were examined in the structural model. All construct-level VIF values were below the recommended threshold of 3.3 (POS - >PD = 1.454, POS - >PS = 1.000, POS - >WLB = 1.000, PS - >PD = 1.475, WLB - >PD = 1.330), suggesting that common method bias is unlikely to represent a substantial threat to the validity and interpretation of the results.

**Figure 2:**
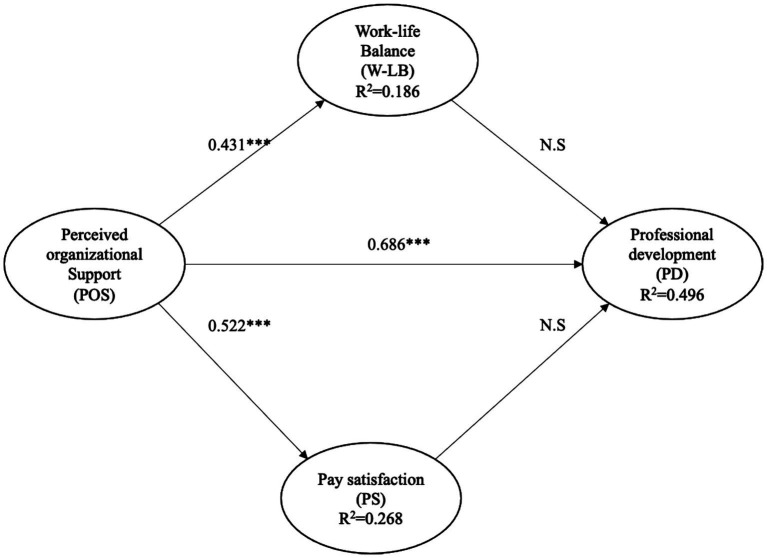
Structural model results.

The coefficients of determination (*R*^2^) indicate that the model explains 49.6% of the variance in Professional Development, 26.8% in Pay Satisfaction, and 18.6% in Work–Life Balance. These results suggest a moderate-to-high explanatory power for the primary dependent variable. The results of the model are shown in Figure 2.

### 4.3 Multigroup analysis

Table 5 presents the generation-specific path coefficients and their statistical significance for Generations X, Y, and Z. Across the three cohorts, Perceived Organizational Support (POS) showed a positive and statistically significant relationship with Professional Development (PD) (Gen X: *β* = 0.703, *p* < 0.001; Gen Y: *β* = 0.717, *p* < 0.001; Gen Z: *β* = 0.627, *p* < 0.001). POS was also positively associated with Work–Life Balance (WLB) for Generations X and Y, although the relationship for Generation Z did not reach conventional levels of significance (*β* = 0.281, *p* = 0.055). Likewise, the relationship between POS and Pay Satisfaction (PS) was significant for Generations X and Y, but not for Generation Z. In contrast, neither WLB nor PS showed significant effects on PD in any of the generational groups.

**Table 5:** Generation-specific path coefficients.

Path	Gen X (*β*)	Gen Y (*β*)	Gen Z (*β*)	*p* value (Gen X)	*p* value (Gen Y)	*p* value (Gen Z)
POS - >WLB	0.548	0.420	0.281	0.000	0.002	0.055
POS - >PD	0.703	0.717	0.627	0.000	0.000	0.000
POS - >PS	0.628	0.542	0.286	0.000	0.000	0.112
WLB - >PD	0.011	0.026	0.112	0.908	0.836	0.490
PS - >PD	0.007	0.021	−0.068	0.944	0.841	0.645

Prior to the multigroup comparisons, measurement invariance was assessed using the MICOM procedure. The results supported compositional invariance for all constructs across the three pairwise comparisons (X–Y, X–Z, and Y–Z), thus establishing partial measurement invariance in all cases. The MICOM results supported compositional invariance for all constructs across the three pairwise comparisons, thereby establishing partial measurement invariance in all cases and supporting the use of permutation multigroup analysis. These results support the use of permutation multigroup analysis to compare the structural relationships across generations.

Table 6 reports the pairwise differences in structural relationships across generations based on the PLS-MGA procedure. The multigroup analysis identified a significant difference only in the relationship between Perceived Organizational Support and Pay Satisfaction, which was stronger for Generation X than for Generation Z (*p* = 0.022). No statistically significant differences were observed in the remaining structural relationships across the generational groups. Given the relatively modest subgroup sizes, particularly for Generation X, these findings should be interpreted with caution.

**Table 6:** Differences in structural relationships across generations.

Path	Diff (Gen X - Gen Y)	Diff (Gen X - Gen Z)	Diff (Gen Y - Gen Z)	*p* value (Gen X vs. Gen Y)	*p* value (Gen X vs. Gen Z)	*p* value (Gen Y vs. Gen Z)
POS - >WLB	0.127	0.266	0.139	0.434	0.087	0.467
POS - >PD	−0.014	0.076	0.090	0.922	0.617	0.526
POS - >PS	0.086	0.342	0.256	0.414	0.022	0.116
WLB - >PD	−0.015	−0.102	−0.086	0.916	0.573	0.664
PS - >PD	−0.013	0.075	0.088	0.943	0.661	0.614

A post-hoc sensitivity assessment indicated that, given the subgroup sizes, the multigroup analysis was better suited to detecting relatively large between-group differences than small or moderate ones. Under conventional assumptions, the minimum detectable effect sizes were in the range of approximately *d* = 0.46 to *d* = 0.55 across the generational comparisons. Accordingly, both the isolated significant difference and the absence of significant differences in the remaining paths should be interpreted cautiously.

Taken together, these findings suggest that Perceived Organizational Support is the primary predictor of Professional Development across cohorts, while the evidence for generational differences in specific structural paths remains preliminary. Overall, Hypothesis 6 received partial support, as a significant difference was observed only for the POS → PS relationship between Generations X and Z, whereas the remaining structural paths did not differ significantly across cohorts.

## 5 Discussion

The findings of this study indicate that Perceived Organizational Support (POS) emerged as the strongest predictor of Professional Development (PD)—operationalized as perceived professional development—particularly in highly demanding, technically intensive industries such as mining in Chile. This empirical evidence is consistent with prior arguments emphasizing that organizational support is associated with the construction of sustainable career trajectories by fostering perceived internal employability, career self-management, and professional commitment (De Vos et al., 2011).

From the perspective of OST (Eisenberger et al., 1986), employees interpret organizational support as a signal of appreciation and recognition, which activates emotional and behavioral reciprocity mechanisms reflected in the intention to stay, improved performance, and career planning efforts. These mechanisms are especially salient in sectors where turnover and shortages of specialized profiles represent structural challenges.

Although the literature has theoretically linked Pay Satisfaction (PS) and Work–Life Balance (WLB) to professional development (Currall et al., 2005; Rajagopal et al., 2024), the present results did not show significant direct effects of either variable on PD, indicating that H4 and H5 were not empirically confirmed. A plausible theoretical explanation is that POS constitutes a more proximal organizational signal of recognition, support, and developmental investment, which aligns closely with how employees evaluate their own professional growth. By contrast, PS and WLB may reflect broader assessments of employment quality that, while important, are not necessarily translated into stronger perceptions of professional development in this sample. In this sense, the findings suggest that employees in the mining sector may interpret institutional support more directly as a resource for growth and advancement, whereas compensation and work–life balance may be appraised primarily in relation to wellbeing, satisfaction, or retention (De Vos and Van der Heijden, 2017; Spurk et al., 2019). This result may also reflect the way these constructs were operationalized in a mining workforce context, an issue that warrants further examination in future studies.

Multigroup analysis suggested a possible difference only in the relationship between POS and Pay Satisfaction, with a stronger association observed for Generation X than for Generation Z. However, this finding should be interpreted with substantial caution given the relatively modest subgroup sizes and the limited statistical power of the multigroup comparisons. Therefore, this result is better understood as preliminary rather than conclusive and should be replicated in larger and more balanced samples before stronger conclusions are drawn regarding cohort-specific differences. If confirmed in future research, with larger samples, it could suggest that older workers are more likely to interpret organizational support through tangible and compensation-related forms of recognition. By contrast, younger cohorts may appraise organizational support less in terms of pay-related perceptions and more in relation to other aspects of the work experience, such as flexibility, development opportunities, or work meaning. Although this study does not directly test those alternative mechanisms, prior research suggests that Generations Y and Z tend to place greater emphasis on continuous learning, work–life balance, and purpose at work (Barhate and Dirani, 2022; Kong et al., 2015). Accordingly, the present findings should be taken as tentative evidence that responses to organizational support may not be fully homogeneous across generational cohorts. However, given the largely non-significant differences observed, this evidence should be considered tentative rather than conclusive.

This study also strengthens the relevance of the sustainable careers perspective, which posits that career success is not limited to hierarchical progression or monetary compensation but also encompasses psychological wellbeing, value alignment, and the capacity to adapt to changing contexts (Spurk et al., 2019). Within this framework, POS appears to function as a structuring resource that may enable employees to navigate complex environments with greater autonomy and motivation.

Finally, this study makes a dual contribution. Theoretically, it provides empirical evidence for a structural relationship that has been examined primarily in service, health, or education settings but remains underexplored in extractive industries. In addition, it provides preliminary evidence that the strength of some organizational relationships is not uniform across generations, thereby extending the discussion on sustainable careers into a multigenerational industrial context. Practically, it offers evidence-based insights suggesting that perceptions of organizational support are more strongly associated with professional development and career planning than pay-related perceptions in this sample. However, these findings should not be interpreted as evidence that support-oriented interventions are necessarily more effective than compensation related policies.

### 5.1 Implications

This study contributes to the literature on professional development by confirming the central role of Perceived Organizational Support (POS) as a direct predictor of sustainable career trajectories, even in technically demanding sectors such as mining. Consistent with the Organizational Support Theory (Eisenberger et al., 1986), the findings reinforce the idea that perceptions of institutional support activate reciprocity mechanisms that strengthen professional development planning, retention, and perceived internal employability.

In addition, the evidence addresses an identified gap by integrating constructs often examined in isolation—POS, Work–Life Balance (WLB), and Pay Satisfaction (PS) (Currall et al., 2005; Masuda et al., 2017). However, only POS emerged as a significant predictor of professional development in the estimated model. This suggests that, in this industrial context, support-oriented organizational signals may be more relevant to employees’ perceptions of professional growth than broader evaluations of compensation or work–life balance. Accordingly, the findings suggest that perceptions of recognition, guidance, and developmental support are more strongly associated with perceived professional development than pay or work–life balance perceptions in this sample.

Moreover, the multigroup analysis offers preliminary evidence that generational cohorts may differ in how organizational support is translated into pay-related perceptions. In particular, Generation X showed a stronger POS–pay satisfaction association than Generation Z; however, this pattern should be interpreted cautiously given the modest subgroup sizes and requires replication before stronger practical conclusions are drawn. This result may tentatively suggest that organizational support is not interpreted identically across career stages and generational cohorts, a possibility that is consistent with prior studies on generational values, work expectations, and career orientations (Barhate and Dirani, 2022; Kong et al., 2015).

Overall, the results support the usefulness of the sustainable careers approach (Spurk et al., 2019) by showing that POS can operate as a key structuring resource for professional development, particularly in environments characterized by high turnover, technical complexity, or strong retention pressures.

From a practical standpoint, the results suggest that Perceived Organizational Support is an important correlate of employee commitment, retention, and professional development in this mining context. The findings suggest that employees who report higher perceived organizational support also report higher perceived professional development. Therefore, organizations may wish to pay attention to development opportunities, explicit recognition, and ongoing institutional support as relevant correlates of professional development in this context.

While compensation and work–life balance conditions remain important for general wellbeing, this study indicates that they are not sufficient on their own to foster sustained professional development. Accordingly, talent management programs may benefit from considering personalized support mechanisms that focus on internal employability, flexible development pathways, and career planning.

In addition, the preliminary generational differences observed in this study suggest the potential value of cohort-tailored approaches in multigenerational industries. While older workers may respond more strongly to tangible incentives and structured recognition, younger cohorts tend to value autonomy, continuous learning, and participatory work environments more intensively. This implies the need to design differentiated professional development strategies aligned with cohort-specific expectations, values, and life stages (Barhate and Dirani, 2022; Kong et al., 2015).

Finally, the use of PLS-SEM and multigroup analysis demonstrates the practical value of evaluating organizational policies and strategically segmenting talent. These tools can support the design and assessment of interventions aimed at professional development and help tailor initiatives to workforce characteristics and needs.

## 6 Conclusion

This study concludes that Perceived Organizational Support (POS) showed the strongest association with Professional Development (PD) in this sample of workers from the Chilean mining sector, whereas Pay Satisfaction (PS) and Work–Life Balance (WLB) were not significantly associated with PD. These findings provide robust empirical evidence within a research domain that remains underexplored in the extractive industries, where high turnover, demanding technical conditions, and the coexistence of multiple generations create distinctive talent management challenges.

From a theoretical perspective, the estimated model supports the continued relevance of Organizational Support Theory (Eisenberger et al., 1986) and its applicability in highly demanding contexts where institutional support becomes a strategic resource for retention, commitment, and professional development. The absence of significant effects of Pay Satisfaction and Work–Life Balance on Professional Development suggests that, in this sample, perceptions of employment quality were less strongly associated with perceived professional growth than perceptions of organizational recognition, support, and developmental investment. This opens opportunities to reconsider how professional development is shaped across industries and job levels, particularly in operational environments, while the evidence regarding generational differences should be regarded as preliminary.

The key limitation of this study is its cross-sectional design, which prevents definitive causal inferences among the analyzed variables. In addition, because all variables were collected through self-reported measures from a single source at one point in time, some risk of common method bias cannot be fully ruled out, even though the full collinearity assessment suggested that it is unlikely to represent a substantial threat to the validity and interpretation of the results. The non-probability convenience sampling approach also limits the generalizability of the findings to the broader mining workforce in Chile. Because recruitment relied on voluntary participation through collaborating organizations, self-selection bias cannot be ruled out. Furthermore, the exact number of employees reached through the dissemination channels was not available, which prevented calculation of a response rate, and no data were available for non-respondents, precluding formal comparisons between respondents and non-respondents. Another relevant limitation is the generational categorization based solely on age ranges, without accounting for individual career trajectories or differentiated social contexts, which may oversimplify the interpretation of the cohort effects. The structural model also did not include control variables such as gender, job role, education, or tenure. Although the model was specified to focus on the theoretically derived relationships among the focal constructs, the absence of such controls means that alternative sources of variation cannot be fully ruled out. The operationalization of Pay Satisfaction and Work–Life Balance, although based on validated scales, may also not have fully captured the specific ways in which these constructs are experienced in the mining context, which should be examined in future research. A further limitation concerns the multigroup analysis. The subgroup sizes, particularly for Generation X, were modest, which reduces statistical power and limits the stability of between-group comparisons. Accordingly, the observed difference in the POS → PS path should be interpreted as preliminary and in need of replication. Future studies should replicate these analyses using larger and more balanced samples, ideally exceeding 100 cases per generational group.

Based on these limitations, several directions for future research are proposed. In particular, future studies should seek to replicate the multigroup comparisons using larger and more balanced generational subsamples to improve statistical power and the reliability of between-group estimates. Future research should also aim to use recruitment strategies that allow better estimation of response rates and potential non-response bias. In addition, future studies should assess the robustness of the proposed relationships by incorporating relevant control variables, such as gender, job role, education, and tenure. First, longitudinal studies could examine how perceptions of organizational support and professional development evolve over time, particularly during technological change or organizational restructuring. Second, future studies may examine additional explanatory variables, such as engagement, self-efficacy, or organizational identification, that could help clarify how POS is associated with professional development. Finally, comparative research across industries or regions could identify differential patterns by organizational type, labor culture, or employment formalization, thereby extending the theoretical and practical applicability of the proposed model.

In summary, this study not only reaffirms the strategic role of POS in complex productive contexts, but also opens new avenues for understanding how organizations can foster professional development processes and sustainable career trajectories while accounting for generational and sector-specific characteristics shaping contemporary work.

## Data Availability

The raw data supporting the conclusions of this article will be made available by the authors, without undue reservation.
